# Comparison of Subjective Refraction under Binocular and Monocular Conditions in Myopic Subjects

**DOI:** 10.1038/srep12606

**Published:** 2015-07-28

**Authors:** Hidenaga Kobashi, Kazutaka Kamiya, Tomoya Handa, Wakako Ando, Takushi Kawamorita, Akihito Igarashi, Kimiya Shimizu

**Affiliations:** 1 Department of Ophthalmology, University of Kitasato School of Medicine, Kanagawa, Japan; 2 Department of Orthoptics and Visual Science, School of Allied Health Sciences, University of Kitasato, Kanagawa, Japan

## Abstract

To compare subjective refraction under binocular and monocular conditions, and to investigate the clinical factors affecting the difference in spherical refraction between the two conditions. We examined thirty eyes of 30 healthy subjects. Binocular and monocular refraction without cycloplegia was measured through circular polarizing lenses in both eyes, using the Landolt-C chart of the 3D visual function trainer-ORTe. Stepwise multiple regression analysis was used to assess the relations among several pairs of variables and the difference in spherical refraction in binocular and monocular conditions. Subjective spherical refraction in the monocular condition was significantly more myopic than that in the binocular condition (p < 0.001), whereas no significant differences were seen in subjective cylindrical refraction (p = 0.99). The explanatory variable relevant to the difference in spherical refraction between binocular and monocular conditions was the binocular spherical refraction (p = 0.032, partial regression coefficient B = 0.029) (adjusted R^2^ = 0.230). No significant correlation was seen with other clinical factors. Subjective spherical refraction in the monocular condition was significantly more myopic than that in the binocular condition. Eyes with higher degrees of myopia are more predisposed to show the large difference in spherical refraction between these two conditions.

At present, in a clinical setting, we measure only subjective refraction, and that, only for monocular testing. However, in man, vision functions under binocular conditions. The evaluation of visual performance under binocular conditions is important. We recently showed that increased pupil diameter under monocular conditions produces higher wavefront aberrations than under binocular conditions^1^. A number of studies have advocated the importance of evaluating the binocular state in post-refractive surgery patients^2^,^3^,^4^,^5^. Subjective refraction forms a fundamental part of the routine optometric eye examination. However, to our knowledge, there have so far been no clinical studies on subjective refraction under binocular conditions.

Overcorrection of eyes by means of lenses or surgery results in headache, eye strain, and eye fatigue^6^,^7^,^8^,^9^. Actually, 30% of patients after refractive surgery are dissatisfied their overcorrection^10^. To prevent overcorrection for ametropia, precise assessment of preoperative subjective refraction is necessary in order to acquire higher patient satisfaction.

The purpose of this study is twofold: to prospectively compare subjective refraction under binocular and monocular conditions, and to investigate the clinical factors that affect the difference in spherical refraction between these two conditions using multivariate regression analysis.

## Results

**Figure 1:**
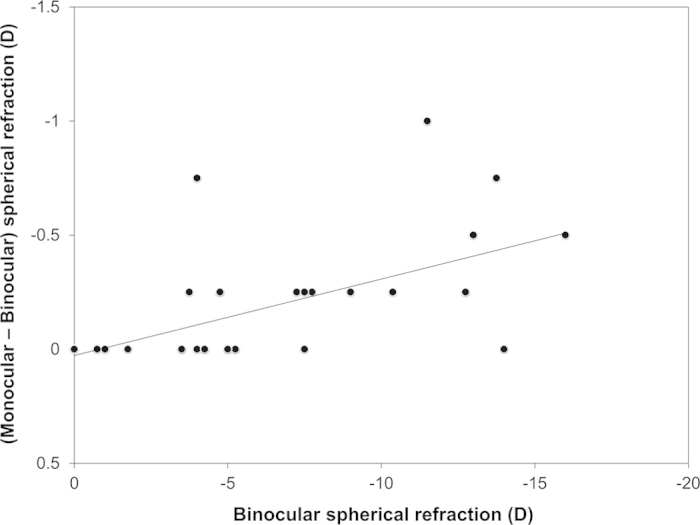
A graph showing a significant correlation between the difference in spherical refraction under binocular and monocular conditions and the binocular spherical refraction (Spearman correlation coefficient r = 0.560, p = 0.001).

The demographics of the study population are shown in Table 1. We found significant differences in spherical refraction (p < 0.001, Wilcoxon’s signed rank sum test) and pupil diameter (p < 0.001) between binocular and monocular conditions, but no significant differences were found in cylindrical refraction (p = 0.999) (Table 2). The mean differences (monocular—binocular) in spherical refraction and pupil diameter were −0.20 ± 0.27 diopter (D) (95% confidence interval (CI), 0.32 to −0.72 D) and 1.40 ± 0.52 mm (95% CI, 0.39 to 2.41 mm), respectively. The results of multiple regression analysis are shown in Table 3. The explanatory variable relevant to the difference in spherical refraction between binocular and monocular conditions was the binocular spherical refraction (p = 0.032, partial regression coefficient B = 0.029) (adjusted R^2^ = 0.230). Multiple regression was expressed by the following equation: difference in spherical refraction between binocular and monocular conditions = (0.029 × binocular spherical refraction) + 0.082. There was no significant correlation shown with other clinical factors such as age, gender, logarithm of the minimal angle of resolution (logMAR) corrected distance visual acuity (CDVA), cylindrical refraction, binocular pupil size, change in pupil size from binocular to monocular conditions, and corneal, or ocular spherical aberration. The standardized partial regression coefficient was calculated in order to determine the magnitude of each variable’s influence. Binocular spherical refraction was the most relevant variable. Similar results were obtained by Spearman’s rank correlation test as shown in Table 3. We excluded the ocular Zernike coefficient of Z 2-0 for a 4-mm pupil from the explanatory variables, because of a multicollinearing between the ocular Zernike coefficient of Z 2-0 and the binocular spherical refraction. The relationship of the difference in spherical refraction between binocular and monocular conditions with the binocular spherical refraction is shown in Fig. 1. With higher degree of myopia, the difference in spherical refraction between binocular and monocular conditions was significantly increased in myopic subjects. Fifteen of 30 eyes (50%) showed greater myopia in their refraction under monocular conditions than under binocular conditions.

**Figure 2:**
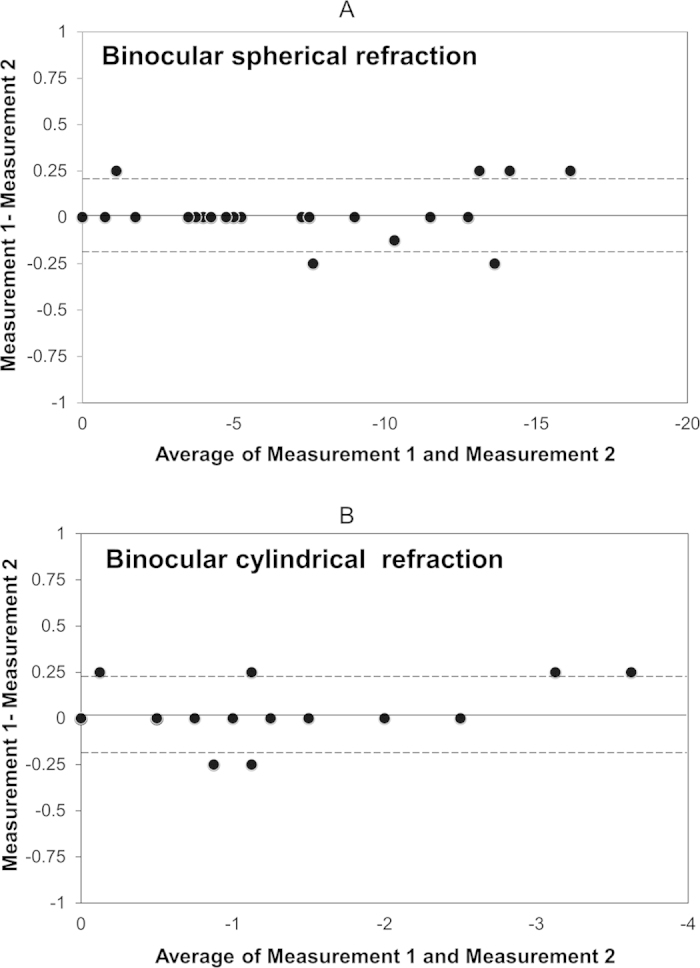
Bland-Altman plots represent the difference between two measurements divided by the mean of these measurements. (**A)** Binocular spherical refraction. (**B)** Binocular cylindrical refraction. The solid lines represent mean differences between 2 measurements of binocular refraction; dotted lines are the upper and lower borders of the 95% limit of agreement (mean difference ± 1.96 multiplied by standard deviation of the mean difference).

Bland-Altman plots indicated that the mean difference between two measurements with binocular refraction (±95% limits of agreement; LoA) was 0.01 ± 0.12 D (−0.21 to 0.24 D) for spherical refraction, 0.08 ± 0.12 D (−0.23 to 0.25 D) for cylindrical refraction (Fig. 2).

## Discussion

In the current study, we demonstrated that subjective spherical refraction under monocular conditions was significantly higher myopic than that under binocular conditions in healthy subjects. However, we found no significant differences in cylindrical refraction between these two conditions. As far as we can ascertain, this is the first published study to compare the subjective refraction under binocular and monocular conditions in healthy subjects. Gwiazda *et al.*^11^ reported that an open-field binocular autorefractor recorded more hyperopia or less myopia than a closed-view monocular autorefractor. The discrepancy in spherical refraction between binocular and monocular conditions might be attributed to differences in pupil sizes under these conditions. The outcomes from the current study also revealed that pupil sizes are larger under monocular viewing conditions than binocular viewing conditions. The larger pupil size may decrease the depth of focus and increase the eye’s blur circle^12^. Accordingly, subjective refraction with a larger pupil may be more myopic than that with a smaller pupil^13^,^14^. However, we found no significant correlation between the difference in spherical refraction under the two conditions and a binocular pupil size or a change in pupil size, presumably because pupil size can be influenced not only by the patient background, such as age^15^, manifest refraction^14^, and the accommodative state of the eye^16^, as well as by various sensory and emotional conditions, but also by the measurement condition affecting the level of retinal illuminance^17^. A further study is needed in order to clarify the exact role of pupil size in the differences of subjective spherical refraction under binocular and monocular conditions.

It has been demonstrated that the average difference of 0.5 to 1.0 mm between monocular to binocular measurements under scotopic and mesopic conditions^18^,^19^,^20^. The difference of pupil size in previous studies was relatively smaller than that of the present study. The discrepancy might be attributed to the differences in measurement conditions, including the illuminance, condition of binocular viewing, and magnification percentage of pupil size.

In the present study, the mean difference in spherical refraction measured by binocular and monocular conditions was not very large (−0.20 ± 0.27 D). However, the eyes up to a maximum of −1.00 D of the difference were observed, indicating that this difference is not negligible in refractive surgery. We assume that overcorrection may occur when myopic error is corrected using only monocular refraction. Eyes overcorrected with lenses or surgery lead to complaints of headache, eye strain, and eye fatigue^6^,^7^,^8^,^9^. It may be necessary to undercorrect myopia when refraction is measured monocularly to prevent overcorrection for myopia. Therefore, it should be noted that the correction of myopia using monocular refraction is not necessarily suitable for refractive surgery. In the correction of myopia, binocular refraction measurement appears to be superior to monocular refraction measurement since the former is performed under natural viewing conditions.

Although spherical refraction alone cannot provide sufficient explanation, as evidenced by the small R^2^ value (R^2^ = 0.230), this lack can affect the difference in spherical refraction between binocular and monocular conditions, suggesting that eyes with higher degrees of myopia are more predisposed to show a large spherical refraction difference in myopic subjects. Accordingly, we should be aware that higher myopia could result in the overcorrection of eyes in refractive surgery when myopia is corrected using only monocular refraction. Although it has been reported that the pupil size in myopia was larger than that in emmetropia^13^,^14^, it still remains unclear why high myopic eyes are more susceptible to show differences between monocular and binocular conditions than low myopic eyes. We presume that eyes with higher degrees of myopia are more predisposed to the effects of their pupil size. A more detailed analysis should be performed to determine the effect of the degree of myopia on the differences in spherical refraction between monocular and binocular conditions in myopia.

It is of clinical importance to assess the repeatability of the measurements with this binocular refraction in order to confirm the applicability of the data. As shown in Fig. 2, we confirmed the good repeatability of the measurements in the current study, as evidenced by the narrow 95% LoA. Hence, we believe that this binocular refraction measurement offers reasonable repeatability in the clinical evaluation of the subjective refraction of the eye.

There are at least three limitations to this study. Firstly, we examined a relatively young group of patients, a group of subjects who frequently have larger pupils, a characteristic that contributes to larger HOAs and higher retinal luminance levels. Most groups of candidates for refractive surgery include these younger subjects. Further study is needed in order to clarify exactly the role of age in binocular and monocular refraction in the eyes of elderly subjects. Secondly, we assessed subjective refraction only in the absence of cycloplegia. We performed a preliminary examination in patients with cycloplegia. Monocular subjective refraction was measured using artificial pupils in patients with cycloplegia. With increasing pupil size, refraction tended to show a higher myopic shift (data not shown). Although we cannot fully deny the possibility that accommodation induces a change in subjective refraction between binocular and monocular conditions, we believe that the presence or absence of accommodation did not alter the subjective refraction in the current study. Thirdly, in the present study, we included only myopic subjects consisting of most of refractive surgery candidates in order to compare subjective refraction under binocular and monocular conditions. Although it still remains unclear whether our results in myopic subjects are on a par with those in hyperopic subjects, this information is clinically meaningful for understanding the etiology of overcorrection after refractive surgery for myopia.

In conclusion, our results demonstrated that subjective spherical refraction under monocular conditions was significantly more myopic than that under binocular conditions in myopic subjects, whereas we found no significant differences in subjective cylindrical refraction. In the correction of myopia, the measurement of binocular refraction appears to be superior to that of monocular refraction in the assessment of natural viewing conditions. Our results also showed that eyes with higher degrees of myopia are more predisposed to showing large differences in spherical refraction between these two conditions.

## Methods

### Subjects

The protocol was registered with the UMIN Clinical Trials Registry (UMIN000015182) at September 16, 2014. Thirty eyes of 30 subjects (16 men and 14 women; mean age ± standard deviation (SD), 29.9 ± 5.5 years) who had no ophthalmic diseases other than refractive errors, were enrolled in this prospective study at Kitasato University Hospital, Kanagawa, Japan. Only the right eyes were tested. The sample size in this study offered 89% statistical power at the 5% level in order to detect a 0.30-D difference in subjective refraction between conditions, when the SD of the mean difference was 0.50 D. The inclusion criteria for this study were as follows: manifest spherical equivalent of −0.50 D or less, when the logMAR CDVA was 0.00 or better, and no exotropia. Eyes with keratoconus were excluded from the study by using the keratoconus screening test of Placido disk videokeratography (TMS-2, Tomey, Nagoya, Japan). The study was approved by the Institutional Review Board at Kitasato University School of Medicine. The methods were carried out in accordance with the approved guidelines. Informed consent was obtained from all subjects after explanation of the nature and possible consequences of the study.

### Refraction Measurements

Subjective refractions without cycloplegia were examined with the 3D visual function trainer-ORTe (3D VFT) (Japan Focus. Co., Ltd, Tokyo, Japan). The 3D VFT is a 3D visual display system for dichoptic viewing. Polarizing glasses with different polarizing filters were used to guarantee that each subject performed monocular use: the one eye but not another able to see the targets. If the filter was designed to exactly match the polarization properties of the 3D monitor, the Landolt-C chart could be perceived by the human eye. If the filter did not match these properties, the eyes could perceive only the backlight without any information. The 3D VFT can display the Landolt-C chart on the monitor. Subjective refraction under binocular conditions was measured through circular polarizing lenses on both eyes. To prevent head-tilt, we used the circular polarizing lens. The tested eye was displayed by the monitor, but the untested eye was not displayed under binocular conditions. Subjects were asked to observe the smallest line of Landolt-C rings they could read binocularly at 5 m with refractive correction. One minute after binocular measurements, monocular refraction was performed by occluding the untested eye. Visual acuity was measured under bright-light conditions (500 lux) and the luminance of a testing target was 130 cd/m^2^ under a circular polarizing lens. All measurements were performed by a single experienced examiner (W. A.). To assess the repeatability of the measurements for confirming the applicability of the data, the measurements with the 3D VFT were made in 30 eyes with binocular refraction at the same time of day on two days. We evaluated the repeatability of the two measurements as described previously using Bland-Altman plots^21^.

### Pupil Measurements

Physiologically dilated horizontal pupil size was measured using the FP-10000 (TMI, Saitama, Japan) infrared electronic pupillometer that was connected to a laptop computer with proprietary pupil analysis software (TMI, version 1.08). The sampling rate was 30 Hz. To assess pupil size in correcting refractive error, we measured pupil size with soft contact lenses on. All measurements were performed under photopic conditions with an ambient illuminance of 500 lux measured using an illuminance meter (T-10, Minolta Corp, Tokyo, Japan). The FP-10000 can measure pupil size in real time under binocular conditions that closely resemble natural viewing conditions^1^,^22^. The magnitude of error introduced by variation in the vertex distance between the cornea and FP-10000 using the circular apertures of 3.0 mm and 5.6 mm was calculated. Ten minutes were allowed for adaptation to the room illuminance prior to the measurements. A crisscross fixation target of 1 degree in the central visual field was placed at a distance of 5.0 m. Under binocular conditions, the pupil diameters of the tested eye were continuously measured for 10 seconds and averaged. Subsequently under monocular conditions, the untested eye was occluded with a black patch, and after two minutes, the pupil diameter of the former eye was again continuously measured for 10 seconds and averaged. We performed the measurement of refraction and pupil size on the same day at the same conditions. The effects of blinking were disregarded.

### Higher-order Aberration Measurements

Corneal and ocular spherical aberrations as Zernike coefficients (Z 2-0 and Z 4-0) for 4-mm and 6-mm pupils were measured by Hartmann-Shack aberrometry (KR-9000PW, Topcon, Tokyo, Japan)^23^,^24^.

### Statistical Analysis

Stepwise multiple regression analysis was performed to investigate the relationships between several variables and the difference in spherical refraction under binocular and monocular conditions. The dependent variable was the difference in spherical refraction under (monocular - binocular) coditions. The explanatory variables included patient age, gender, logMAR CDVA, refraction (sphere and cylinder) under binocular conditions, binocular pupil size, change in pupil size from binocular to monocular conditions, and corneal or ocular spherical aberrations. Spearman’s rank correlation test was also performed to assess the relationships of this difference in spherical refraction with other variables. Since normal distribution of the data was not confirmed with the Kolmogorov-Smirnov test (p < 0.001), the Wilcoxon signed-rank test was used to compare the binocular and monocular data. The results are expressed as mean ± standard deviation, and a p-value of <0.05 was considered statistically significant.

## Additional Information

**How to cite this article**: Kobashi, H. *et al.* Comparison of Subjective Refraction under Binocular and Monocular Conditions in Myopic Subjects. *Sci. Rep.* **5**, 12606; doi: 10.1038/srep12606 (2015).

## Figures and Tables

**Table 1:** Demographics of the study population

	**Patient Demographics**	
Age (years)	29.9 ± 5.5 years (95% CI, 19.1 to 40.7 years)	
Gender (Male : Female)	M : F = 16 : 14	
LogMAR CDVA	−0.17 ± 0.05 (95% CI, −0.26 to −0.08)	
	4-mm pupil	6-mm pupil
Corneal spherical aberration		
Z 2-0	−0.59 ± 0.06 μm (95% CI, −0.71 to −0.47 μm)	−0.91 ± 0.27 μm (95% CI, −1.44 to −0.37 μm)
Z 4-0	0.04 ± 0.02 μm (95% CI, 0.00 to 0.08 μm)	0.19 ± 0.11 μm (95% CI, −0.02 to 0.39 μm)
Ocular spherical aberration		
Z 2-0	3.85 ± 1.95 μm (95% CI, 0.03 to 7.67 μm)	8.64 ± 4.17 μm (95% CI, 0.46 to 16.82 μm)
Z 4-0	0.02 ± 0.03 μm (95% CI, −0.05 to 0.08 μm)	0.07 ± 0.19 μm (95% CI, −0.29 to 0.44 μm)

CI = confidence interval, logMAR = logarithm of the minimal angle of resolution, CDVA = corrected distance visual acuity.

**Table 2:** Subjective refraction and pupil diameter under binocular and monocular conditions

**Measurement**	**Binocular**	**Monocular**	**Difference (Monocular-Binocular)**	**P value**
Spherical refraction (D)	−6.80 ± 4.38 (95% CI, 1.80 to −15.39)	−7.00 ± 4.54 (95% CI, 1.89 to −15.89)	−0.20 ± 0.27 (95% CI, 0.32 to −0.72)	< 0.001
Cylindrical refraction (D)	−0.81 ± 0.91 (95% CI, 0.98 to −2.59)	−0.81 ± 0.91 (95% CI, 0.98 to −2.59)	0.00	0.999
Pupil diameter (mm)	3.51 ± 0.57 (95% CI, 2.39 to 4.63)	4.91 ± 0.65 (95% CI, 3.64 to 6.18)	1.40 ± 0.52 (95% CI, 0.39 to 2.41)	< 0.001

D = diopter, CI = confidence interval.

**Table 3:** Results of correlation analysis and stepwise multiple regression analysis to select variables relevant to the difference in subjective refractions under binocular and monocular conditions

**Variables**	**Spearman correlation coefficient**	**P value**	**Partial regression coefficient**	**Standardized partial regression coefficient**	**P value**
Age (years)	−0.145	0.444	not included		—
Gender (male = 0, female = 1)	−0.059	0.757	not included		—
LogMAR CDVA	−0.095	0.618	not included		—
Spherical refraction (D)	0.560	0.001	0.029	0.474	0.032
Cylindrical refraction (D)	0.350	0.058	not included		—
Binocular pupil size (mm)	0.148	0.437	not included		—
Change in pupil size (mm)	−0.099	0.604	not included		—
Corneal spherical aberration (μm)					
Z 2-0 for a 4-mm pupil	0.254	0.060	not included		—
Z 4-0 for a 4-mm pupil	0.104	0.128	not included		—
Ocular spherical aberration (μm)					
Z 4-0 for a 4-mm pupil	0.163	0.217	not included		—
			0.082	Constant	Adjusted R^2^ = 0.230

logMAR = logarithm of the minimal angle of resolution, CDVA = corrected distance visual acuity, D = diopter.
